# Annealing Effect on Linear and Ultrafast Nonlinear Optical Properties of Bi_2_Te_3_ Thin Films

**DOI:** 10.3390/ma17246281

**Published:** 2024-12-22

**Authors:** Tengfei Zhang, Shenjin Wei, Xiaoxiao Song, Shubo Zhang, Yaopeng Li, Yiyun Zou, Ying Wang, Menghan Li, Ying Jiang, Junhua Wang, Ertao Hu, Jing Li

**Affiliations:** 1 Department of Optical Science and Engineering, Shanghai Ultra-Precision Optical Manufacturing Engineering Center, Fudan University, Shanghai 200433, China; 21110720020@m.fudan.edu.cn (T.Z.); shejin_wei@fudan.edu.cn (S.W.); 19110720013@fudan.edu.cn (X.S.); 20110720017@fudan.edu.cn (S.Z.); 19110720073@fudan.edu.cn (Y.L.); 21210720017@m.fudan.edu.cn (Y.Z.); 22110720013@m.fudan.edu.cn (Y.W.); 23110720009@m.fudan.edu.cn (M.L.); 22210720001@m.fudan.edu.cn (Y.J.); 2 Shanghai Frontiers Science Research Base of Intelligent Optoelectronics and Perception, Institute of Optoelectronics, Fudan University, Shanghai 200438, China; wangjunhua@fudan.edu.cn; 3 College of Electronic and Optical Engineering and Jiangsu Province Engineering Research Center for Fabrication and Application of Special Optical Fiber Materials and Devices, Nanjing University of Posts and Telecommunications, Nanjing 210023, China; iamethu@njupt.edu.cn

**Keywords:** Bi_2_Te_3_ thin film, magnetron sputtering method, annealing treatment, Z-scan, third-order optical nonlinearities

## Abstract

In recent years, the fabrication of materials with large nonlinear optical coefficients and the investigation of methods to enhance nonlinear optical performance have been in the spotlight. Herein, the bismuth telluride (Bi_2_Te_3_) thin films were prepared by radio-frequency magnetron sputtering and annealed in vacuum at various temperatures. The structural and optical properties were characterized and analyzed using X-ray diffraction, scanning electron microscopy, X-ray photoelectron spectroscopy, spectroscopic ellipsometry, and UV/VIS/NIR spectrophotometry. The third-order optical nonlinearities of Bi_2_Te_3_ thin films were investigated using the Z-scan technique, employing a 100 fs pulse width at an 800 nm wavelength. It is found that the crystallinity and the average grain size of the films increase with the annealing temperature. Meanwhile, the extinction coefficient of the annealed films increased, accompanied by a redshift in the optical bandgap. All samples exhibit pronounced saturable absorption and self-focusing behaviors. The nonlinear absorption coefficient and nonlinear refractive index of Bi_2_Te_3_ films annealed at 300 °C were found to be 2.44 times and 1.85 times higher than those of the as-deposited films, respectively. These findings demonstrate that annealing treatment is an effective approach to tuning the crystalline structure and linear optical properties of Bi_2_Te_3_ films while simultaneously enhancing their nonlinear optical performance.

## 1. Introduction

Recently, there has been growing interest in nonlinear optical absorption (NOA) materials that play a significant role in mode-locking fiber lasers [1], super-resolved direct laser writing [2], all-optical diodes [3], optical limiting [4], and other fields owing to their high NOA coefficient and large laser damage threshold. According to the different electron transition mechanisms, the NOA process can be divided into saturable absorption (SA) and reverse saturable absorption (RSA) processes connected with the Stokes transition and anti-Stokes transition, respectively [5]. In general, the NOA process and coefficient are closely related to the excitation wavelength and optical band gap of semiconductor materials. Based on first principles, the topological properties and surface states of third-generation topological insulators, represented by Bi_2_Te_3_, have been investigated and demonstrated [6]. Due to its small bulk optical bandgap and unique topological insulator characteristics, Bi_2_Te_3_ exhibits broadband optical saturable absorption properties across the 800–1550 nm wavelength range [7]. Furthermore, the novel light modulation with high chemical and thermal stability has been successfully developed, showing great potential for applications in passive mode-locked lasers [8]. The application of new NOA materials in ultrafast optics can manipulate the shape and intensity of pulsed lasers, which provides a new idea for improving the performance of optical devices.

Bi_2_Te_3_ is a member of the V_2_VI_3_ binary chalcogenide compound semiconductor family. There have been a lot of studies on its thermoelectric properties due to its high thermoelectric figure of merit and Seebeck coefficient, as early as the last century [9]. However, the research on the third-order nonlinear optical properties of Bi_2_Te_3_ started late. In 2012, Bernard et al. first reported the SA behavior of Bi_2_Te_3_ at a 1550 nm wavelength [10]. Then, Zhao et al. claimed that the 95% modulation depth at 1550 nm was found in the Bi_2_Te_3_ nanosheet, and it could be applied to mode-locked lasers [11]. In 2012, Molli et al. observed RSA response in nanocrystalline Bi_2_Te_3_ under nanosecond laser excitation at 532 nm [12]. He et al. successfully fabricated nanoscale Bi_2_Te_3_/PMMA composite films, which demonstrated high saturable intensity and a modulation depth of 15.1% under 130 fs pulses at a wavelength of 800 nm [13]. In 2019, Qiao et al. investigated and observed strong two-photon absorption effects in layered Bi_2_Te_3_ samples through Z-scan measurement using femtosecond laser pulses at 1056 nm [14]. In recent decades, more and more research and attention have been carried out in the field of NOA materials in order to enhance the NOA properties. For Bi_2_Te_3_, doping foreign elements and forming heterojunctions with other materials are effective methods to improve its nonlinear absorption properties. For instance, Molli et al. demonstrated that thallium-doped Bi_2_Te_3_ exhibited enhanced nonlinear response in comparison with pristine Bi_2_Te_3_ due to the increase of free carrier density [12]. Wang et al. fabricated a graphene/Bi_2_Te_3_ heterojunction structure that possesses much higher modulation depth and accelerates inter-band carrier recombination [15]. Despite these insights, the methods to improve the nonlinear optical absorption and refraction properties of Bi_2_Te_3_ thin films have yet to be fully explored. It is well known that annealing treatment can release the internal stress within the film, improve the crystallinity, and subsequently influence the microstructure. This approach has been demonstrated to be an effective method for improving the quality of the film. Therefore, it can be reasonably proposed that annealing represents another potential tool for the tuning of the physical and optical properties of Bi_2_Te_3_ films.

In this work, Bi_2_Te_3_ thin films with appropriate thicknesses were prepared by the magnetron sputtering method, and the crystal structure, transmittance, refractive index, extinction coefficient, and other parameters of Bi_2_Te_3_ thin films are obtained through a series of methods after thermal treatment in vacuum conditions. We further explore the third-order nonlinear optical properties of prepared Bi_2_Te_3_ films at different annealing temperatures using the open aperture (OA) and close aperture (CA) Z-scan technique. The results of this study are practicable for applying the Bi_2_Te_3_ thin films with tunable thermal treatment in ultrafast nonlinear optical fields.

## 2. Materials and Methods

The Bi_2_Te_3_ thin films were deposited on both Si (100) and fused quartz substrates by the radio-frequency (RF) magnetron sputtering system (EBAS, Infovion, Bucheon-si, Republic of Korea) using a 99.999% purity Bi_2_Te_3_ target at room temperature. Each substrate was cleaned with acetone, alcohol, and deionized water successively in an ultrasonic cleaner and dried with high-purity nitrogen. Before deposition, the pre-sputtering was conducted for 10 min to remove contamination from the target surface and make the sputtering rate tend to be steady. The sputtering power was set to 50 W in RF mode, and the sputtering time was fixed at 120 s. The background and working pressures of deposition in the chamber are controlled at 4.5 × 10^−3^ mTorr and 3.6 mTorr, respectively. The sputtering process was conducted in a high-purity argon environment, maintained at a flow rate of 40 standard cubic centimeters per minute. Generally, the thin films obtained by magnetron sputtering are usually amorphous in nature. After deposition, the amorphous Bi_2_Te_3_ films prepared under the same conditions were annealed at 150 °C, 200 °C, 250 °C, and 300 °C, respectively, in a vacuum condition (lower than 0.1 Pa) for 1 h. According to different annealing temperatures, the film samples were labeled as S150, S200, S250, and S300, respectively.

X-ray diffraction (XRD) with Cu-Kα (λ = 1.54056 Å) radiation (Bruker D8 ADVANCE, Karlsruhe, Germany) was used to analyze the crystal structure of Bi_2_Te_3_ thin films. The diffraction range (2θ) was set from 10° to 60° with a step of 0.02°. The section thickness of samples was observed by scanning electron microscopy (SEM) (ZEISS Gemini300, Jena, Germany) at a 3 kV acceleration voltage. The element mapping and the chemical composition were studied by energy-dispersive X-ray spectroscopy (EDS) attached to the SEM, and bonding analysis was carried out by X-ray photoelectron spectroscopy (XPS) (Thermo Scientific K-Alpha, Waltham, MA, USA). The linear optical constants, such as refractive index *n* and extinction coefficient *k*, were obtained by spectroscopic ellipsometry (SE). UV/VIS/NIR spectrophotometer (Shimadzu UV-3600, Kyoto, Japan) was employed to measure transmission spectra of Bi_2_Te_3_ thin films in the wavelength range of 300–1500 nm.

The third-order nonlinear optical properties of Bi_2_Te_3_ thin films were tested by a single-beam Z-scan system that was first proposed by M. Sheik-bahae in 1989 [16]. The laser resource was a Ti:sapphire regenerative amplifier system (Spectra Physics, Mountain View, CA, USA). The laser wavelength, pulse width, and repetition rate are 800 nm, 100 fs, and 1 kHz. The output power of the laser can be precisely controlled by two attenuators. The focal length of the convex lens was 30 cm, and the beam radius *ω_0_* at the focal point was about 46.5 ± 1.2 μm by fitting the experimental data. It could be seen that the film thickness in this study was much smaller than the Rayleigh length ${(z}_{0}{=\pi \omega }_{0}^{2}/\lambda )$, which could be considered as “thin” film [17]. The system was calibrated using standard CS_2_ solution in a 1 mm thick quartz cuvette.

## 3. Results and Discussion

### 3.1. XRD Analysis

**Figure 1:**
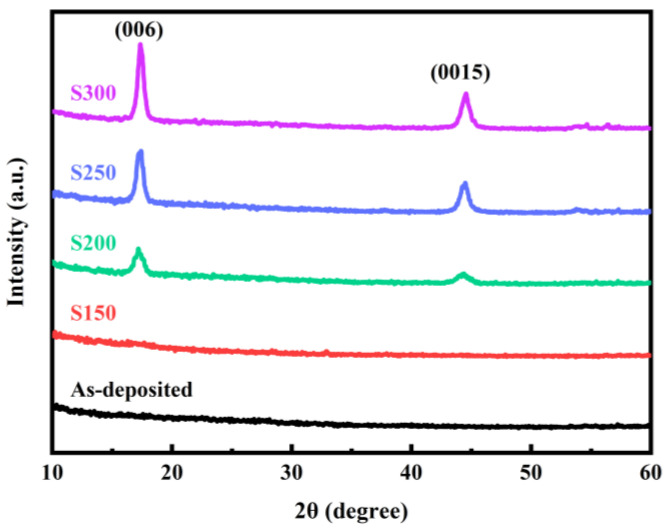
XRD patterns of Bi_2_Te_3_ thin films annealed at different temperatures.

The X-ray diffraction patterns of Bi_2_Te_3_ thin films deposited on Si (100) substrates are shown in Figure 1. The annealing treatment provides additional energy to the atoms within the thin films, facilitating atomic migration and rearrangement. This leads to a phase transition from the amorphous to the crystalline state, enhancing the crystallinity and reducing lattice defects and strain. The absence of structural peaks for as-deposited and S150 samples exhibits the amorphous nature. This can be attributed to the low annealing temperature, which is inadequate to provide the requisite heat energy to overcome the energy barrier associated with atomic diffusion and rearrangement. Diffraction peaks are observed in films annealed at temperatures above 200 °C, indicating their polycrystalline characteristics. As the annealing temperature increases, the Bragg diffraction peaks become sharper, and their intensity increases, demonstrating an improvement in the crystallinity of the Bi_2_Te_3_ thin films. The typical diffraction peaks are indexed to (006) and (0015) crystal planes, which are consistent with the rhombohedral crystal of Bi_2_Te_3_ (JCPDS NO: 15-0863) with lattice parameters a = b = 4.385 Å and c = 30.483 Å (Space group: R3m) [18].

Based on the Scherrer equation [19], the mean crystallite size (*D*) can be estimated from the XRD peak width of (006):

(1) $$D=\frac{0.94\lambda }{B\cos\theta }$$

where *λ* = 1.54056 Å is the radiation of the XRD, *B* is the full width at half maximum (FWHM) of the highest diffraction peak, which can be gotten from Figure 1, and *θ* is half of the angle between the incident and the scattered X-ray beams. The density of the dislocation (*δ*) and micro-strain (*ε*) in the films is determined by the following expression:

(2) $$\delta =\frac{C}{D^{2}}$$

(3) $$\varepsilon =\frac{B\cos\theta }{4}$$

where C is a factor equal to 1, which represents the minimum dislocation density. The average size of the crystallite calculated by theoretical calculation increases from 10.13 nm to 13.22 nm as the annealing temperature increases from 200 °C to 300 °C, while the density of the dislocation and micro-strain decreases. Similar results have been reported in previous literature [20], which are attributed to the release of residual stress, enhancement of clusters, and rearrangement of atoms [21]. The evidence presented here demonstrates that thermal annealing has an impact on the transformation of the material under examination from amorphous-to-crystalline. An increase in the annealing temperature results in an enhancement in the crystallinity of the film. A comprehensive summary of the detailed structural parameters associated with the (006) peak can be found in Table 1.

### 3.2. SEM, EDS, and XPS Analysis

**Figure 2:**
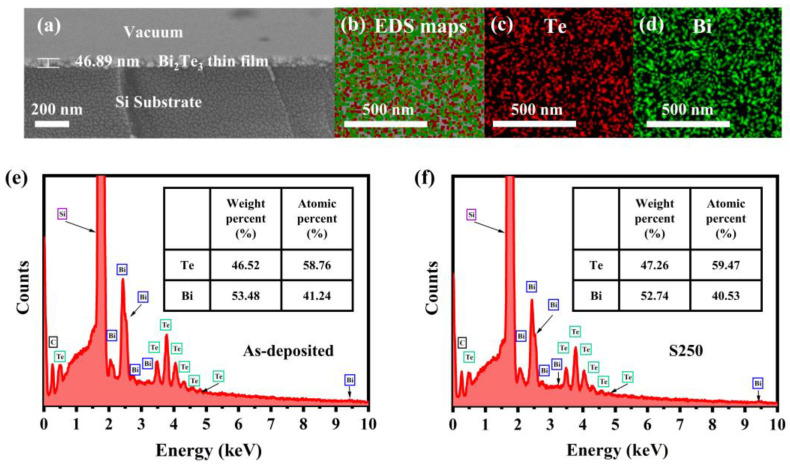
The cross-sectional SEM image of (**a**) the S250 sample; (**b**–**d**) element mapping images of the S250 sample for Te (red dots) and Bi (green dots); EDS spectrums of (**e**) the as-deposited and (**f**) the S250 sample.

From the SEM image displayed in Figure 2a, the estimated film thickness of S250 is approximately 47 nm. In the SEM image, the vacuum environment, Bi_2_Te_3_ layer, and Si substrate are clearly distinguished. To investigate the elemental composition and prove the quality of the thin film, the samples were characterized by EDS. As shown in the elemental maps of Figure 2b–d, the uniformity and continuity of the distribution of Bi and Te elements are demonstrated in which the green and red dots represent elements Te and Bi, respectively.

The elemental weight and atomic percent of as-deposited (Figure 2e) and S250 (Figure 2f) samples were calculated to further investigate the change in film composition before and after annealing. It is evident that the main elements of the samples are Si, Bi, and Te, as shown in Figure 2e,f. The element Si comes from the silicon substrate, and the Bi and Te are derived from the film. The atomic ratio of Bi to Te in the as-deposited sample is about 0.70, while the atomic ratio of Bi to Te in the S250 sample is 0.68, which is closer to the stoichiometric ratio of 0.67 in standard Bi_2_Te_3_. This indicates that annealing treatment can promote the transformation of the disordered structure in the film into an ordered crystal structure, increase the crystallinity of the film (as shown in Figure 1), and reduce the defects in the film, such as grain boundaries, vacancies, and interstitial atoms, thereby stabilizing the elemental ratio.

**Figure 3:**
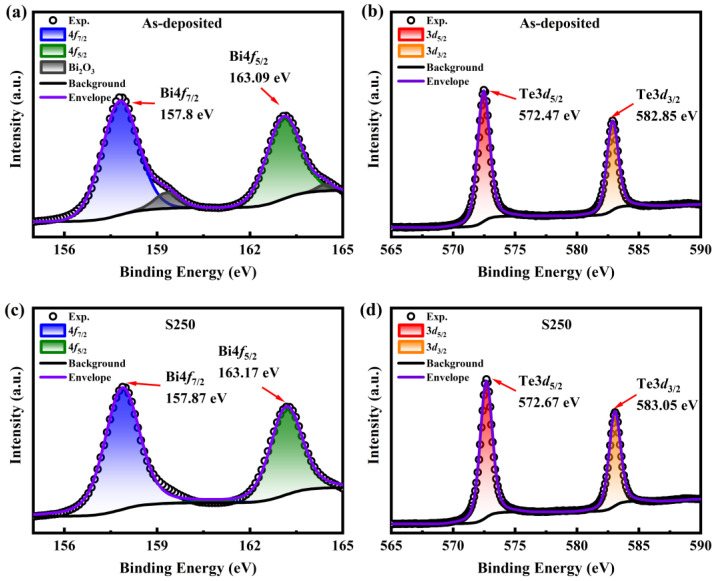
XPS spectrums of (**a**) Bi 4f (**b**) Te 3d for the as-deposited and (**c**) Bi 4f (**d**) Te 3d for the S250 samples.

XPS is a non-destructive spectroscopic surface analysis technique that can measure the valence state and provide a semi-quantitative estimate of each element on the surface of the sample. The XPS spectrum was corrected using the C1s peak at 284.8 eV binding energy to compensate for the effect of surface charges [22]. The XPS spectra of bismuth and tellurium for the as-deposited sample are presented in Figure 3a,b, while those for the S250 sample are shown in Figure 3c,d. For the as-deposited sample, two peaks are observed at 157.8 eV and 163.09 eV, which correspond to Bi 4f_7/2_ and Bi 4f_5/2_, respectively, as shown in Figure 3a. Through data fitting, additional peaks at 159.38 eV and 164.48 eV are identified in it, which can be attributed to the Bi/O bond in Bi_2_O_3_ caused by surface oxidation of Bi_2_Te_3_ film [22]. In fact, the Bi 4f_7/2_ peak consists of two contributions located at 157.8 eV and 159.38 eV, while the corresponding Bi 4f_5/2_ peaks are located at 163.09 eV and 164.48 eV, which correspond to Bi in Bi_2_Te_3_ and Bi in Bi_2_O_3_, respectively. Following vacuum annealing for the S250 sample, as shown in Figure 3c, only two peaks were observed at binding energies of 157.87 eV and 163.17 eV, implying that Bi exists in the annealed Bi_2_Te_3_ thin film in the form of Bi^3+^. This indicates that during the annealing process, the O element separates from the film surface, preventing the formation of a Bi/O bond. The elimination of Bi/O bonds maintains the stoichiometric ratio of the thin film, contributing to the formation of a more stable crystalline structure. Additionally, it prevents the formation of uneven surface oxidation layers, which could otherwise lead to localized stress and defects. Moreover, the spectrum shows two strong peaks at 572.47 eV and 582.85 eV in Figure 3b, which can be assigned to Te 3d_5/2_ and Te 3d_3/2_, attributed to the Te^2-^ state with a spin-orbit splitting of 10.38 eV. The peaks from Te/O are not noted for both the as-deposited and the S250 samples in Figure 3b,d. XPS spectra confirmed the formation of the Bi_2_Te_3_ phase in the films. The results of the EDS and XPS suggest that annealing treatment may contribute to a ratio of element Bi to Te that is closer to 2/3. The annealing treatment is beneficial to optimize the quality and performance of the films and reduce the potential negative effects caused by oxidation, thereby improving their optical properties and stability.

### 3.3. Linear Optical Analysis

**Figure 4:**
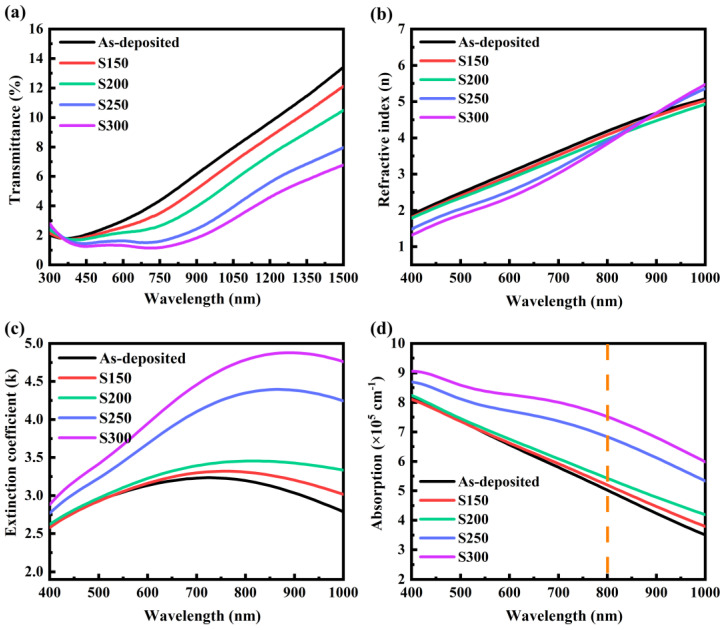
The (**a**) transmission spectrum, (**b**) *n*, (**c**) *k* curves, and (**d**) absorption spectrum of Bi_2_Te_3_ thin films.

The transmission spectrum of Bi_2_Te_3_ thin films measured by UV-3600 is displayed in Figure 4a. Obviously, the transmittance of all samples is less than about 5% in the visible light region, which exhibits a semi-metallic nature for Bi_2_Te_3_ [19], and the transmittance gradually increases from the visible light region to the near-infrared light region. In addition, the sample annealed at a higher temperature has correspondingly lower spectral transmittance, and the absorption edge moves to a longer wavelength (red shift), indicating that the optical bandgap becomes narrow. This may be owing to the process of amorphous to crystalline transition of the Bi_2_Te_3_ thin film caused by annealing [23]. It can be seen from the figure that the transmittance of the annealed Bi_2_Te_3_ thin film in the visible band is extremely low, and the transmittance changes gently. By controlling the annealing temperature, it is possible to adjust the transmittance of the Bi_2_Te_3_ thin films within a certain range, which significantly increases the potential applications of these thin films in devices for the attenuation of visible radiation.

Other essential parameters, such as film thickness (*d*), refractive index (*n*), and extinction coefficient (*k*), are measured by SE and accurately obtained by modeling and data fitting [24]. The fitting thicknesses of the deposited and thermally treated films S150, S200, S250, and S300 are 45.04 nm, 45.02 nm, 46.7 nm, 46.82 nm, and 46.04 nm, respectively. The discrepancy between the thickness determined by fitting and the cross-sectional thickness assessed by SEM is approximately ± 1 nm. The results of *n* and *k* obtained by fitting the SE data are depicted in Figure 4b,c. Both *n* and *k* for Bi_2_Te_3_ thin films are very high, which indicates a large linear absorption coefficient and imaginary part of the third-order polarizability. Therefore, this material has potential applications in third-order optical nonlinearity, especially in the NOA field [25]. The value of *k* at wavelengths between 400–1000 nm exhibited an appreciable enhancement as the temperature of annealing increased. These alterations result in an enhanced absorption of the film in the VIS/NIR region and a reduction in transmittance. This is mainly because the crystallinity of the film increases after annealing treatment, and the grain size of the film increases with the annealing temperature, which leads to quite strong scattering loss [26]. From this figure, it can be seen that the red shift of the peak of the *k* curve, as well as the transmission spectrum, indicates the decrease in the optical band gap. The values of *n* and *k* before and after crystallization show significant distinctions in the figure, which corresponds to the crystallization results observed in the XRD.

The linear absorption coefficient (*α*) can be obtained by the relationship with *k* in the following formula:

(4) α=4πk/λ

As shown in Figure 4d, the *α* of the Bi_2_Te_3_ thin films exceeds 10^5^ cm^−1^ from the visible to the near-infrared region, which indicates a pronounced light-matter interaction. The changes in microstructures and defects gradually strengthen the optical linear absorption of the films with the increase in annealing temperature at the wavelength of 800 nm. It is widely accepted that annealing treatment represents an effective method for enhancing the crystallinity of semiconductor films and eliminating unsaturated defects. In our experiment, due to the complexity of the internal structural defects of the chalcogenide film, there are still a large number of unsaturated defects in the forbidden band of the crystallized film that exert a significant influence on the absorption of photons. In the case of nanostructure films, the existence of defects can significantly enhance the diversity of properties, while the variation of the defect state during the annealing process can regulate optical absorption behaviors.

**Figure 5:**
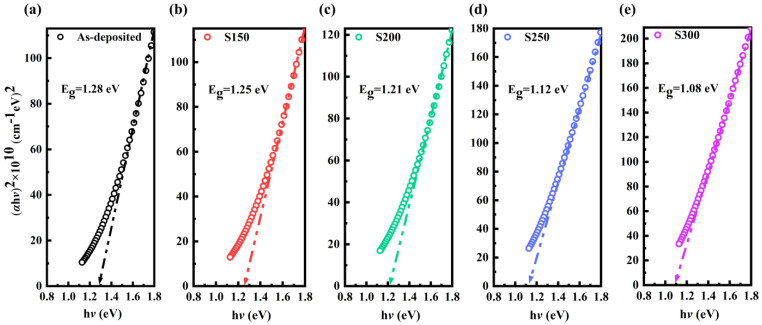
The optical band gap of Bi_2_Te_3_ thin films: (**a**) as-deposited, (**b**) S150, (**c**) S200, (**d**) S250, (**e**) S300.

Based on the Tauc equation [27], the optical band gap (*E_g_*) of the Bi_2_Te_3_ thin film can be obtained by using *α* and *λ*:

(5) $$\alpha h\nu =C{\left(h\nu -E_{g}\right)}^{n}$$

where C is a constant related to the effective mass, h is the Planck constant, h*ν* refers to the incident photon energy, and *n* is a certain value depending on the type of transition. According to the report by Adam, *n* is equal to 1/2 for direct transitions in Bi_2_Te_3_ thin films [19]. The variation of (*α*h*ν*)^2^ versus h*ν* is given in Figure 5. The data are linearly fitted and extended to the *x*-axis, and the intercept at the *x*-axis is the *E_g_*. Obviously, the result that *E_g_* decreases from 1.28 eV to 1.08 eV with increasing annealing temperature is consistent with the red shift of transmittance and extinction coefficient curves. This behavior can be explained in the Davis–Mott model during the process of amorphous–crystalline transformation [28]. Under thermal annealing conditions, sufficient vibration energy can break some weaker bonds, thus introducing some dangling bonds around the surface of the crystallites during the crystallization process. These dangling bonds are responsible for some types of defects in the highly polycrystalline solids [29]. Consequently, the concentration of localized states in the band structure increases, resulting in a decrease in the optical band gap. The reduction in the *E_g_* with increasing annealing temperatures has also been observed in some other chalcogenide thin films such as Sb_2_Se_3_ [30], Bi_5_Ge_40_Se_55_ [23], and Ga_15_Se_83_In_2_ [31]. The linear optical constants of the films obtained above provide essential data support for the subsequent analysis of NOA properties.

It was found that by annealing at different temperatures, the transmittance, extinction coefficient, absorption coefficient, and optical band gap of Bi_2_Te_3_ thin films change regularly. The results suggest that annealing treatment is an effective method to modulate the linear optical properties of Bi_2_Te_3_ thin films.

### 3.4. Nonlinear Optical Analysis

**Figure 6:**
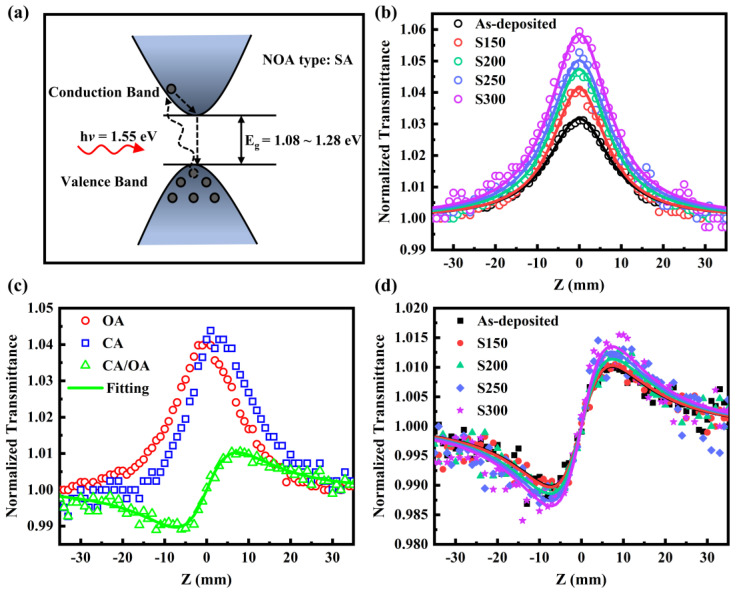
(**a**) Electronic transition diagram of SA process and (**b**) OA, (**c**) CA, and (**d**) divide CA by OA Z-scan curves of the Bi_2_Te_3_ thin films.

A single-beam Z-scan technique with femtosecond laser pulses at an 800 nm wavelength was carried out to investigate the third-order nonlinear optical properties of Bi_2_Te_3_ thin films. The relatively low incident intensity (*I*_0_) of 33 GW/cm^2^ was selected to avoid the nonlinear signal of quartz substrates and nonlinear scattering of samples. The OA Z-scan curves of the Bi_2_Te_3_ thin films are shown in Figure 6b, where the dots are experimental data, and solid lines are the fitting results. It can be seen that the normalized transmittance gradually increases as the film moves toward the focal point, which exposes the nonlinear SA response for Bi_2_Te_3_ thin films. The typical SA behavior can be interpreted by the relationship between the *E_g_* (1.28 eV–1.08 eV) and the photon energy of the laser source (1.55 eV). Since the incident photon energy is much larger than *E_g_*, the electrons in the valence band are excited to transition to the conduction band, as simplified in Figure 6a, leading to the conduction band being almost occupied. After that, Pauli-blocking avoids further absorption of photons, resulting in SA response, which is also an effective explanation for most semiconductor samples. All the Z-scan curves are symmetrical to the focal point (*z* = 0), and meanwhile, this procedure is reversible, indicating that the SA property of the Bi_2_Te_3_ thin film is laser-induced nonlinearity rather than originating from the phase transition of samples [32]. The SA becomes much more pronounced, represented by larger modulation depth with the increasing annealing temperature.

The fitting of OA Z-scans using Equation (6) was employed to determine the NOA coefficient (*β*) related to the SA [17].

(6) $$T_{\mathrm{OA}}\left(z\right)=\overset{\infty }{\underset{m=0}{\sum }}\frac{{\left[{-\beta I}_{0}L_{\mathrm{eff}}/{(1+z}^{2}/z_{0}^{2})\right]}^{m}}{{\left(m+1\right)}^{3/2}}$$

Here, *T_OA_* is the normalized transmittance, *I*_0_ is the peak intensity of the incident laser at the focus, and *z*_0_ stands for the Rayleigh length of the beam, which is much larger than the thickness of each sample. Furthermore, *L_eff_* is the effective thickness of samples defined by Equation (7), which can be calculated by the linear absorption coefficient (*α*_0_) at 800 nm and film thickness (*l*).

(7) $$L_{\mathrm{eff}}=\frac{\left({1-e}^{{-\alpha }_{0}l}\right)}{\alpha_{0}}$$

Note that the imaginary part of third-order nonlinear optical susceptibility (Im*χ*^(3)^) is directly related to *β* by the following equation:

(8) Imχ(3)=[(10−7cλn2)96π2]β

where Im*χ*^(3)^ is in esu, *β* is in cm/W, and c is the speed of light in vacuum. A figure of merit (FOM) for the third-order optical nonlinearity is defined as FOM=|Imχ(3)/α0| to eliminate the discrepancy caused by the linear absorption, which can be analyzed in the SA performance of the Bi_2_Te_3_ thin films in this study.

All the NOA parameters for the Bi_2_Te_3_ thin films are listed in Table 2. Compared with the amorphous films, the *β* of the crystalline films is larger, and the |*β*| gradually increases from (1564.85 ± 22.19) cm/GW to (3815.47 ± 42.27) cm/GW with increasing annealing temperature. The NOA coefficient of Bi_2_Te_3_ thin films in this study is one order of magnitude larger than that of other chalcogenide films, such as MoS_2_ [33], WS_2_ [33], and Sb_2_Se_3_ [34]. And it is similar to that of Bi_2_Se_3_ [35] with the same rhombohedral structure. Much work so far has focused on appropriate values of *β* materials for ultrashort pulse generation [36] and passive photonic diodes [37] with a high nonreciprocity factor. For this reason, annealing treatment can be an efficient method to improve performances in ultrafast nonlinear optics.

The CA Z-scan experiments are performed to ascertain both the sign and magnitude of nonlinear optical refraction (NOR). Figure 6c shows OA, CA, and CA/OA Z-scan curves of the Bi_2_Te_3_ thin films. Due to the influence of strong SA, the CA curve also exhibits a peak structure, but the peak is slightly shifted away from the center position. In order to eliminate the impact of the nonlinear optical absorption effect, the normalized transmittance collected by the CA Z-scan is divided by the OA data. The pre-focal valley and the post-focal peak imply a positive sign for the nonlinear refractive index, which suggests the presence of a self-focusing effect within the Bi_2_Te_3_ film under 800 nm fs laser pulse irradiation. This finding is consistent with the previously reported results for Bi_2_Te_3_ nanosheets [38]. The CA/OA curve is fitted by the following formula:

(9) $$T_{\mathrm{CA}/\mathrm{OA}}\left(z\right)=1+\frac{{4x\Delta \varphi }_{0}}{{(x}^{2}{+9)(x}^{2}+1)}$$

(10) $${\Delta \varphi }_{0}{=kn}_{2}L_{\mathrm{eff}}I_{0}$$

where *x* = *z*/*z*_0_ and Δ*φ*_0_ is the on-axis nonlinear phase shift at the focus. As the annealing temperature is increased, the value of the NOR index (*n*_2_) rises from (1.07 ± 0.03) × 10^−2^ cm^2^/GW to (1.98 ± 0.05) × 10^−2^ cm^2^/GW. The *n*_2_ of Bi_2_Te_3_ thin films is on the same order of magnitude as that reported for Bi_2_Te_3_ nanosheets [38] and is two orders of magnitude higher than that of MoTe_2_ [39]. The distance between the maximum and minimum normalized transmittances of the studied samples is about 1.7*Z*_0_, which is consistent with the phenomenon observed for Kerr nonlinearity. This indicates that the source of NOR is mainly the third-order NOR caused by bound electrons at low intensities of laser radiation [16]. In the present case of a femtosecond pulse width and a low repetition rate laser, the nonlinear response caused by the thermal effect can be considered to be almost negligible. Re*χ*^(3)^ in esu can be derived through the equations [40]:

(11) Reχ(3)=n02c12π2n2 × 10−7

where *n*_2_ is in cm^2^/W, *n*_2_ and Re*χ*^(3)^ obtained for Bi_2_Te_3_ thin films under femtosecond pulse excitation are given in Table 2.

The nonlinear optical absorption coefficient and nonlinear refractive index of Bi_2_Te_3_ thin films increased with annealing temperature, confirming that annealing treatment is an effective method for enhancing third-order nonlinear optical properties. The nonlinear optical parameters of the Bi_2_Te_3_ thin films are compared with those of other advanced two-dimensional materials and topological insulator materials, as shown in Table S1.

**Figure 7:**
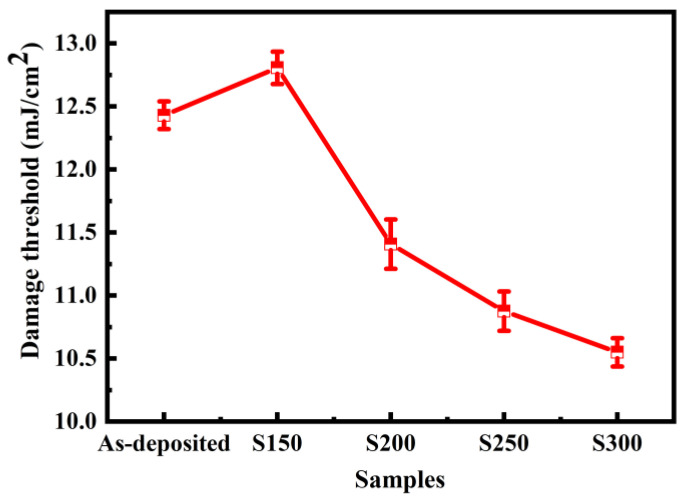
Damage thresholds of annealing treatment Bi_2_Te_3_ thin films.

Under the irradiation of high-energy lasers, the damage threshold of thin films is one of the most important factors to limit the performance in high-power laser systems. For application purposes, the damage threshold of Bi_2_Te_3_ films was investigated. As denoted in Figure 7, the maximum damage threshold is (12.81 ± 0.13) mJ/cm^2^ at an annealing temperature of 150 °C, but it decreases with the further increase in annealing temperature. The laser-induced damage threshold of optical thin films depends on several factors, including light absorption, surface morphology, microstructure, thermal and mechanical properties, etc. After undergoing low-temperature annealing, the thermal conductivity of the film is enhanced through the crystallization process, which effectively leads to an increase in the damage threshold [41]. At higher annealing temperatures, the main reason for the decrease in the damage threshold is the increase in the absorption coefficient of the film (as shown in Figure 4d) [42]. Both high modulation depth and large damage threshold are the key parameters for mode lockers. Accordingly, annealing treatment plays a critical role in the modulation of Bi_2_Te_3_ thin films with excellent third-order nonlinear optical performances.

## 4. Conclusions

In summary, Bi_2_Te_3_ thin films were prepared by RF magnetron sputtering, and then the as-deposited thin films were annealed in vacuum at different temperatures (150–300 °C with an interval of 50 °C). The effects of annealing on the microstructure, linear optical properties, and third-order nonlinear optical responses of Bi_2_Te_3_ thin films were investigated in detail. The as-deposited Bi_2_Te_3_ thin films exhibited an amorphous structure, which transformed into a polycrystalline phase when annealed at temperatures above 200 °C. Annealing treatment can improve the crystallinity of Bi_2_Te_3_ thin films and inhibit the formation of Bi/O bonds, thus stabilizing the element ratio. Enhanced crystallinity led to an increase in the linear absorption coefficient and a decrease in the calculated *E_g_* from 1.28 eV to 1.08 eV. Meanwhile, the transmittance gradually decreases with annealing temperature in the spectral range of 400–800 nm and remains relatively constant across this wavelength region, making it a potential optical attenuator for the visible band. The third-order nonlinear optical properties of Bi_2_Te_3_ thin films were examined using OA and CA Z-scan systems, revealing a dominant nonlinear absorption effect. Compared to other chalcogenide thin films, the Bi_2_Te_3_ thin films exhibited remarkably strong SA performance under femtosecond excitation, with annealing further enhancing the *β* and *n_2_*. The fitted |*β*| for the as-deposited films was (1564.85 ± 22.19) cm/GW, which increased to (3815.47 ± 42.27) cm/GW after annealing, correlating with the reduced *E_g_* and improved crystallinity. This study demonstrates that vacuum annealing is a highly effective method for enhancing the nonlinear optical properties of Bi_2_Te_3_ thin films while also tuning their crystalline structure and optical characteristics. These properties make Bi_2_Te_3_ thin films promising candidates for saturable absorbers in applications such as mode-locked lasers, Q-switching, all-optical diodes, and so on. By taking advantage of its tunable optical properties, the most suitable parameters can be obtained within a certain range, which contributes to the enhancement of ultrafast optical device performance.

## Figures and Tables

**Table 1:** FWHM, average crystallite sizes, dislocation density, and micro-strain of Bi_2_Te_3_ thin films.

Samples	FWHM (°)	*D* (nm)	*δ* (10^15^ × lines/m^2^)	*ε* (×10^−3^)
S200	0.83 ± 0.02	10.14 ± 0.23	9.74 ± 0.44	3.57 ± 0.08
S250	0.69 ± 0.01	12.13 ± 0.13	6.80 ± 0.14	2.98 ± 0.03
S300	0.64 ± 0.02	13.23 ± 0.37	5.73 ± 0.32	2.74 ± 0.08

**Table 2:** NOA parameters of the Bi_2_Te_3_ thin films measured by Z-scan.

Samples	*L_eff_* (nm)	*β* (cm/GW)	*n*_2_ (×10^−2^ cm^2^/GW)	Im*χ*^(3)^ (×10^−9^ esu)	Re*χ*^(3)^ (×10^−9^ esu)	FOM (×10^−14^ esu × cm)
As-deposited	17.84 ± 0.12	−1564.85 ± 22.19	1.07 ± 0.03	−6.95 ± 0.11	4.77 ± 0.15	1.38 ± 0.02
S150	17.37 ± 0.14	−1816.49 ± 34.64	1.13 ± 0.07	−7.69 ± 0.15	4.78 ± 0.29	1.48 ± 0.03
S200	16.96 ± 0.09	−2242.74 ± 26.51	1.30 ± 0.04	−8.94 ± 0.11	5.19 ± 0.16	1.65 ± 0.02
S250	14.04 ± 0.07	−3026.03 ± 20.73	1.69 ± 0.04	−11.77 ± 0.08	6.56 ± 0.17	1.72 ± 0.01
S300	12.88 ± 0.11	−3815.47 ± 42.27	1.98 ± 0.05	−14.23 ± 0.16	7.39 ± 0.19	1.89 ± 0.02

## Data Availability

Data are contained within the article.

## Supplementary Materials

The following supporting information can be downloaded at: https://www.mdpi.com/article/10.3390/ma17246281/s1, Table S1: Comparison of the nonlinear optical parameters for different materials. Reference [43] are cited in the supplementary materials.
