# Supplementary material for: Annealing Effect on Linear and Ultrafast Nonlinear Optical Properties of Bi2Te3 Thin Films
Source: Materials (Basel). 2024 Dec 22;17(24):6281. doi: 10.3390/ma17246281 (PMC11728218; doi:10.3390/ma17246281)
Supplement: Supplementary file 1 [file materials-17-06281-s001.zip › materials-3373229-supplementary.pdf]

materials

MDPI

Supplementary material

Annealing Effect on Linear and Ultrafast Nonlinear Optical Properties of Bi_2Te_3 Thin Films

Tengfei Zhang [1], Shenjin Wei [1], Xiaoxiao Song [1], Shubo Zhang [1], Yaopeng Li [1], Yiyun Zou [1], Ying Wang [1], Menghan Li [1], Ying Jiang [1], Junhua Wang [2], Ertao Hu [3], and Jing Li [1,*]

1 Department of Optical Science and Engineering, Shanghai Ultra-Precision Optical Manufacturing Engineering Center, Fudan University, Shanghai 200433, China; 21110720020@m.fudan.edu.cn (T.Z.); shejin_wei@fudan.edu.cn (S.W.); 19110720013@fudan.edu.cn (X.S.); 20110720017@fudan.edu.cn (S.Z.); 19110720073@fudan.edu.cn (Y.L); 21210720017@m.fudan.edu.cn (Y.Z.); 22110720013@m.fudan.edu.cn (Y.W.); 23110720009@m.fudan.edu.cn (M.L.); 22210720001@m.fudan.edu.cn (Y.J.)
2 Shanghai Frontiers Science Research Base of Intelligent Optoelectronics and Perception, Institute of Optoelectronics, Fudan University, Shanghai 200438, China; wangjunhua@fudan.edu.cn
3 College of Electronic and Optical Engineering and Jiangsu Province Engineering Research Center for Fabrication and Application of Special Optical Fiber Materials and Devices, Nanjing University of Posts and Telecommunications, Nanjing 210023, China; iamethu@njupt.edu.cn
* Correspondence: lijing@fudan.edu.cn; Tel.: +86-021-65643439

Table S1. Comparison of the nonlinear optical parameters for different materials.

Materials	Laser	β (cm/GW)	n_2 (cm²/GW)	$\chi^{(3)}$ (×10⁻⁹ esu)	Reference
MoS_2	1030 nm, 1 kHz, 340 fs	-250 ± 50	-	-	[33]
WS_2	800 nm, 1 kHz, 40 fs	-397 ± 40	-	-	[33]
Sb_2Se_3	800 nm, 1 kHz, 100 fs	-7.82×10^2	-	-	[34]
Bi_2Se_3	800 nm, 2 kHz, 35 fs	-1.67×10^3	0.18	-	[35]
$MoTe_2$	1064 nm, 10 kHz, 100 ps	-2.99 ± 0.52	$(-0.16 \pm 0.027) \times 10^{-3}$	9.5×10^{-12}	[39]
Graphene	800 nm, 1 kHz, 100 fs	$-1.52 \pm 0.42\times10^{-2}$	-	-	[39]
Graphene	1064 nm, 10 kHz, 100 ps	-	-13.7×10^{-3}	-	[39]
Bi_2Te_3	800 nm, 1 kHz, 100 fs	-	0.097	-	[38]
Bi_2Te_3	532 nm, 4 ns	-	-	1.52×10^{-10}	[43]
Bi_2Te_3	800 nm, 1 kHz, 100 fs	-1564.85 ± 22.19 ~ -3815.47 ± 42.27	$(1.07 \pm 0.033) \times10^{-2}$ ~ $(1.98 \pm 0.051) \times10^{-2}$	$(8.42 \pm 0.15) \times10^{-9}$ ~ $(16.03 \pm 0.23) \times10^{-9}$	This work

References

33. Zhang, S.; Dong, N.; McEvoy, N.; O'Brien, M.; Winters, S.; Berner, N. C.; Yim, C.; Li, Y.; Zhang, X.; Chen, Z., Direct observation of degenerate two-photon absorption and its saturation in WS_2 and MoS_2 monolayer and few-layer films. *ACS Nano* **2015,** 9, 7142-7150.

34. Liu, C.; Cheng, L.; Yuan, Y.; Su, J.; Zhang, X.; Li, X.; Zhao, H.; Zhang, H.; Zheng, Y.; Li, J., Contrastive investigation on linear optical properties and nonlinear absorption behaviors between Sb_2Se_3 and Sb_2Te_3 thin films. *Mater. Res. Express* **2019,** 6, 086446.

35. Xiao, S.; Fan, Q.; Ma, Y.; Zhao, Q.; Wang, G.; Xin, H.; Qin, Y.; Yu, X.; He, J., Reversal in optical nonlinearities of Bi_2Se_3 nanosheets dispersion influenced by resonance absorption. *Opt. Express* **2019,** 27, 21741-21749.

38. Miao, L.; Yi, J.; Wang, Q.; Feng, D.; He, H.; Lu, S.; Zhao, C.; Zhang, H.; Wen, S., Broadband third order nonlinear optical responses of bismuth telluride nanosheets. *Opt. Mater. Express* **2016,** 6, 2244-2251.

39. Wang, K.; Feng, Y.; Chang, C.; Zhan, J.; Wang, C.; Zhao, Q.; Coleman, J. N.; Zhang, L.; Blau, W. J.; Wang, J., Broadband ultrafast nonlinear absorption and nonlinear refraction of layered molybdenum dichalcogenide semiconductors. *Nanoscale* **2014,** 6, 10530-10535.

43. Hurtado-Aviles, E. A.; Trejo-Valdez, M.; Torres, J. A.; Ramos-Torres, C. J.; Martínez-Gutiérrez, H.; Torres-Torres, C., Photo-induced structured waves by nanostructured topological insulator Bi_2Te_3. *Opt. Laser Technol.* **2021**, 140, 107015.
